# Global Burden of Colorectal Cancer in Adolescents and Young Adults From 1990 to 2021: A Systematic Analysis of the Global Burden of Disease Study 2021

**DOI:** 10.1002/hsr2.71587

**Published:** 2025-11-30

**Authors:** Ya‐Fei He, Lu Zhang, Dun‐Zhong Hu, Chang‐Zheng Li, Dan Zhang, Yan‐Ni Li, Ning‐Bo Hao, Ling Zhao

**Affiliations:** ^1^ Department of Intensive Care Medicine Beijing Zhongguancun Hospital Beijing China; ^2^ Department of Anesthesiology Zibo Central Hospital Zibo China; ^3^ Department of Emergency Medicine PLA Rocket Force Characteristic Medical Center Beijing China; ^4^ Department of Gastroenterology PLA Rocket Force Characteristic Medical Center Beijing China; ^5^ Health Management Section PLA Rocket Force Characteristic Medical Center Beijing China; ^6^ Department of Anesthesiology PLA Rocket Force Characteristic Medical Center Beijing China

**Keywords:** adolescents and young adults, colorectal cancer, global burden of disease, incidence, mortality

## Abstract

**Background and Aims:**

Colorectal cancer (CRC) is a major global health concern, with rising early‐onset cases (EOCRC) among adolescents and young adults (AYAs, 15–39 years). This study evaluates the global burden, trends, and risk factors of AYA CRC from 1990 to 2021 using Global Burden of Disease (GBD) 2021 data.

**Methods:**

Utilizing GBD 2021 estimates, we analyzed incidence, prevalence, mortality, disability‐adjusted life years (DALYs), and attributable risk factors. To analyze temporal trends, we employed joinpoint regression with a maximum of four nodes, and future burden projections were generated using ARIMA models. Analyses were stratified by age, sex, region, and Socio‐Demographic Index (SDI).

**Results:**

Global AYA CRC incidence rose from 41,384 cases in 1990 to 70,201 in 2021 (AAPC: 0.35), with the highest age‐standardized incidence rate (ASIR) in East Asia (5.11 per 100,000). Mortality decreased (AAPC: −1.00), driven by improved healthcare, while prevalence increased. Low whole‐grain intake (15.9% DALYs) and high red meat consumption (12.7%) dominated globally, while high BMI emerged as a growing contributor, particularly in high‐SDI regions. Males had higher burden than females, peaking at ages 35–39. By 2060, the ASIR is projected to rise by 14.7%, with the most pronounced increases in low‐middle SDI regions, where healthcare infrastructure remains underdeveloped.

**Conclusion:**

The rising incidence of AYA CRC underscores urgent needs for targeted prevention, early screening, and lifestyle interventions addressing modifiable risks like diet and obesity. Geographic and socioeconomic disparities highlight the importance of equitable healthcare access to mitigate future burden.

## 1 Introduction

Colorectal cancer (CRC) is the third most commonly diagnosed cancer and the second leading cause of cancer‐related deaths worldwide [1]. In 2020, approximately 147,950 individuals were diagnosed with CRC and 53,200 died from the disease [2]. Although traditionally associated with older adults, recent decades have witnessed a marked rise in early‐onset colorectal cancer (EOCRC), particularly among adolescents and young adults (AYAs) aged 15–39 years [3, 4]. This demographic shift poses significant challenges, as AYAs remain typically excluded from routine CRC screening programs, leading to delayed diagnoses and advanced‐stage disease at presentation [5, 6]. The underlying drivers of this trend are still poorly characterized, necessitating urgent epidemiological investigations to guide prevention and early detection strategies.

The Global Burden of Disease (GBD) study offers critical insights into age‐specific CRC patterns. While CRC incidence has declined in older populations in high‐income countries (HICs) due to screening initiatives, GBD data reveal a striking 2% annual increase in AYA cases since the 1990s, with pronounced geographic disparities [7]. For instance, high Socio‐Demographic Index (SDI) regions, including North America and Western Europe, report the steepest rises, contrasting with stable or declining rates in low‐SDI areas such as sub‐Saharan Africa [8]. These variations suggest complex interactions between genetic susceptibility, environmental exposures, and lifestyle changes, though systematic analyses focusing on AYAs are limited.

Healthcare access disparities further complicate the situation. AYA CRC tends to be a more advanced disease at presentation than CRC in older patients [9]. Factors such as limited healthcare provider awareness deficits, cultural stigma, and fragmented health systems exacerbate diagnostic delays, contributing to poorer outcomes in younger populations [10]. Despite growing recognition of AYA CRC, research has predominantly focused on individuals aged 40–49 years, leaving a critical knowledge gap for those aged 15–39.

This study aims to evaluate the trends in CRC among adolescents and young adults aged 15–39 years using the most recent GBD 2021 data. We examine the global epidemiology of CRC, highlighting trends and variations across factors such as age, sex, region, and SDI. Additionally, we identify key risk factors contributing to the rise of AYA CRC.

## 2 Methods

The study was conducted in accordance with the Declaration of Helsinki and approved by the Institutional Ethics Committee of PLA Rocket Force Characteristic Medical Center, China (KY2025038).

### 2.1 Data Source

The study utilized estimates from GBD 2021 study (publicly available at https://vizhub.healthdata.org/gbd-results/) for all the analyses. The GBD‐2021 study estimated mortality and causes of death for 371 diseases and injuries, along with 88 risk factors or combinations thereof, across 204 countries and territories with 811 subnational locations from 1990 to 2021. The 204 countries and territories were further categorized into seven super‐regions and 21 regions for more detailed analysis and comparison. Data sources of GBD‐2021 study included censuses, household surveys, civil registration and vital statistics, disease registries, health service utilization, sensors/monitors, satellite imaging, etc. The methodology has been published previously [11, 12, 13].

### 2.2 Estimation of Disease Burden

AYA CRC was arbitrarily defined as cases occurring in individuals from 15 to 39 years due to CRC based on ICD‐9 and ICD‐10 codes (https://ghdx.healthdata.org/record/ihme-data/gbd-2021-cause-icd-code-mappings). In this study, all estimates produced by sex (male and female), age (5 age groups from 15 to 39 years), and year (annually between 1990 and 2021) are presented as the mean estimates accompanied by 95% uncertainty intervals (UIs), which were the 2.5th and 97.5th percentile values across 500 random draws [12]. The prevalence, incidence, disability‐adjusted life years (DALYs), years lived with disability (YLDs), years of life lost (YLLs) as well as the mortality rates, were each reported in terms of the number of cases or events per 100,000 individuals on an annual basis. Age‐standardized rates and corresponding 95% confidence intervals (CI) were calculated using the GBD standard population structure [11].

The correlation of CRC burden in relation to country‐level development, as quantified by the SDI was further examined. The SDI is a composite metric derived from lag‐distributed income per capita, the average educational attainment of individuals aged 15 and above, and the total fertility rate among those younger than 25. The final SDI value was calculated as the geometric mean of three components, scaled ranging from 0 (representing the lowest value) to 100 (the highest), based on country‐specific data. The GBD study divided the 204 countries and territories into five categories according to their SDI values: those with a low SDI (< 0.45), low‐middle SDI (ranging from 0.45 to < 0.61), middle SDI (from 0.61 to < 0.69), high‐middle SDI (between 0.69 and < 0.80), and high SDI (≥ 0.80).

### 2.3 Risk Factors

The GBD study determined the risk factors based on a comparative risk assessment framework, which consisted of six sequential steps [14, 15]. Specifically, attributable DALYs refers to the decrease in current disease DALYs that could occur if the population's exposure to the risk factor shifted to a different or counterfactual distribution. It was calculated by multiplying the total DALYs of a particular outcome by the population attributable fraction (PAF). The PAF represents the portion of outcomes that would decline in a specific population and time if the exposure reached the theoretical minimum risk exposure level's counterfactual value. For CRC, the GBD‐2021 study provides three level‐1 risk factors: environmental/occupational factors, metabolic factors, and behavioral risk factors. We determined 11 level‐2 risk factors for AYA CRC, which were low‐calcium diet, low‐fiber diet, low‐milk diet, low‐whole‐grain diet, high‐processed‐meat diet, and high‐red‐meat diet, high fasting plasma glucose, high body mass index (BMI), low physical activity, high alcohol use, and smoking. Notably, due to the inherent limitations of the GBD 2021 dataset—which only provides attribution estimates of individual risk factors to DALYs rather than raw data on joint exposures—we were unable to analyze interaction effects between risk factors (e.g., the synergistic effect of “high red meat intake + low fiber intake”) in the current study.

### 2.4 Statistical Analysis

The study utilized R software (version 4.1.0) for all the data preparation, statistical analyses and figure plotting, unless otherwise described. A *p* value of smaller than 0.05 (two‐sided) was regarded as statistically significant.

For assessing trends of the DALY over time, joinpoint regression, also called as log‐linear model segmented regression analysis, was performed using the Joinpoint application (Version 5.1.0, National Cancer Institute of the United States) [16, 17]. Average annual percentage changes (AAPCs) and annual percentage change (APC) and their 95% CIs determined using a linear regression model were used to quantify the trends. The maximal nodes were set to four during the analysis.

The disease burden of AYA CRC until 2060 were predicted using the autoregressive (AR) integrated moving average (ARIMA) (p, d, q) model [18, 19, 20]. The model comprises the AR model and moving average (MA) model. The values of p, d, and q denote the orders of autoregression, the degree of difference, and the order of MA, respectively. The ARIMA equation is $Y_{t}=\alpha +\phi_{1}Y_{t-1}+\phi_{2}Y_{t-2}+\cdots +\phi_{p}Y_{t-p}+\varepsilon_{t}+\theta_{1}\varepsilon_{t-1}+\cdots +\theta_{q}\varepsilon_{t-q}.$ $\phi_{a}\left(a=1,2,\cdots ,p\right)$ and $\theta_{b}\left(b=0,1,2,\cdots ,q\right)$ are the parameters of the AR and MA, respectively. *Y*_*t*_ represents the starting value, and *ε*_*t*_ is the value of the random error at time *t*. *α is the intercept and is a constant*. The whole process involves four steps. First, the functional form of the model was determined. Then, the appropriate parameters were selected. The third and fourth step involves model verification and prediction, respectively. The Augmented Dickey‐Fuller (ADF) test was used to check for stationarity of the time series. The autocorrelation (ACF) and partial autocorrelation (PACF) functions helped determine the optimal model parameters (*p* and *q*) which were selected based on the Akaike Information Criterion (AIC) and Bayesian Information Criterion (BIC). The Ljung‐Box Q test was then utilized to verify that normal distribution the residuals of the chosen models.

## 3 Results

### 3.1 AYA CRC Incidence

**Figure 1:**
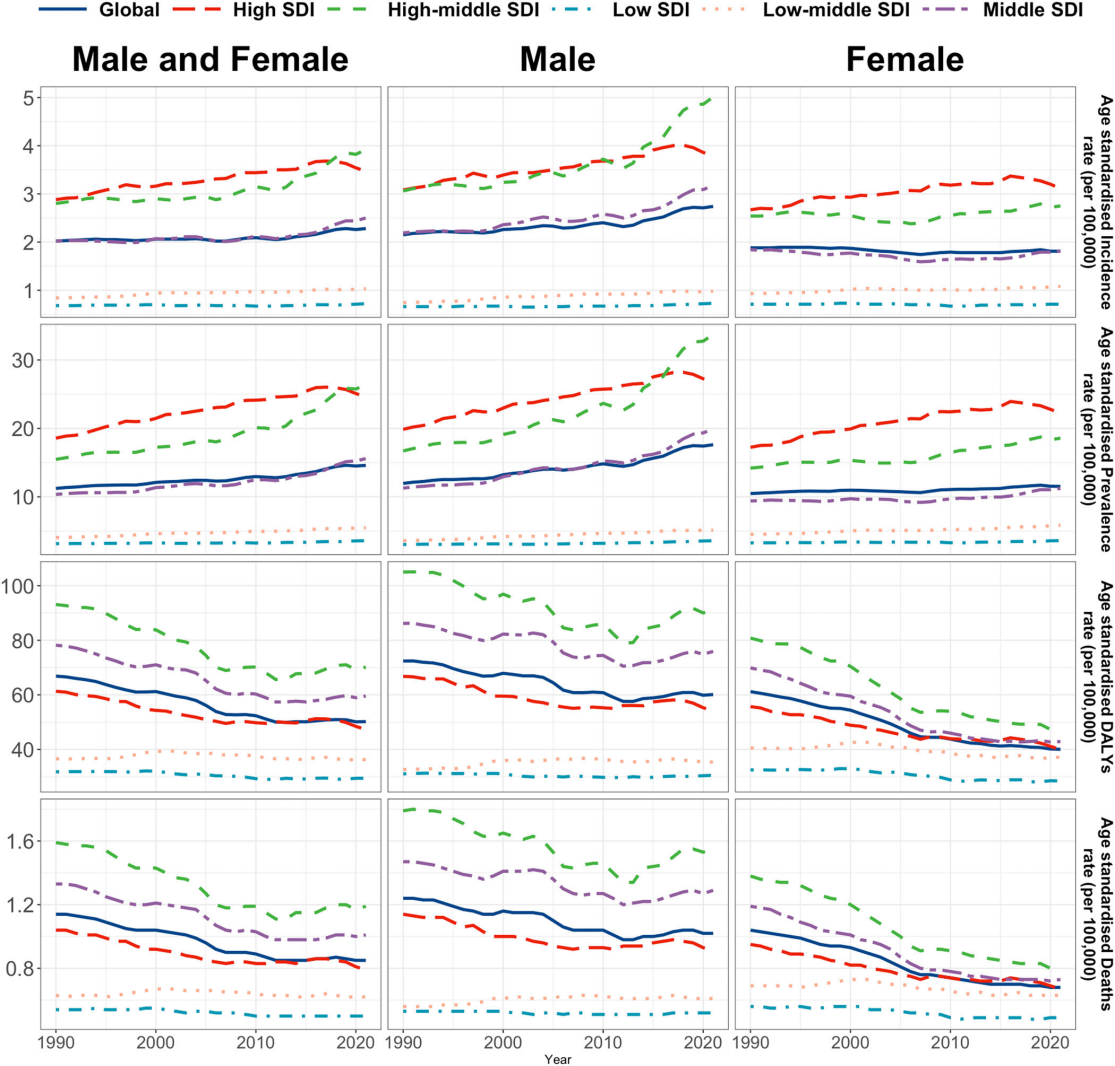
Temporal trend of age standardized incidence rate, age standardized prevalence rate, age standardized disability adjusted life years (DALYs) rate, and age standardized mortality rate for the burden of AYA CRC from 1990 to 2021.

**Figure 2:**
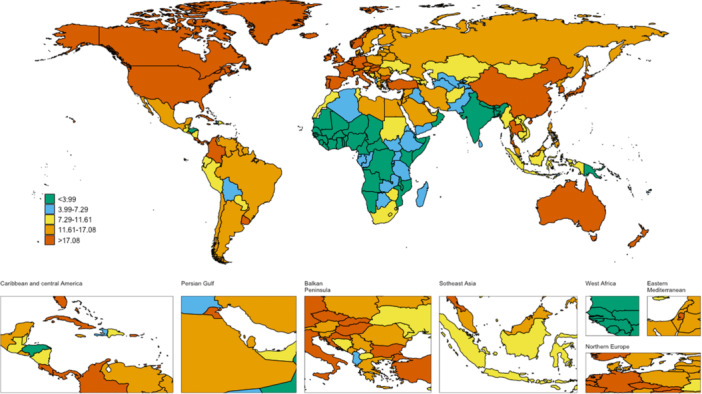
The global disease burden of AYA CRC incidence rate for both sexes in 204 countries and territories.

Globally, the number of AYA CRC cases increased from 41,384 (95% CI: 37,653–44,500) in 1990 to 70,201 (95% CI: 64,050–76,855) in 2021. This rise was accompanied by an increase in the age‐standardized incidence rate (ASIR), from 2.02 (95% CI: 2.00–2.04) to 2.28 (95% CI: 2.26–2.30) per 100,000 individuals, yielding an AAPC of 0.35 (95% CI: 0.10–0.61). The incidence of AYA CRC increased in all five SDI regions, with the most pronounced rise in the high‐middle SDI category, showing an AAPC of 1.01 (95% CI: 0.54–1.48). In 2021, the highest ASIR was observed in the High‐middle SDI group (3.92; 95% CI: 3.86–3.97) (Figure 1 and Table 1). Geographically, AYA CRC incidence rose in all 21GBD regions, with East Asia having the highest ASIR at 5.11 (95% CI: 5.05–5.17), followed by Australasia at 4.36 (95% CI: 3.99–4.76) in 2021. From 1990 to 2021, the largest increases in AYA CRC incidence were observed in Central Latin America, with an AAPC of 2.44 (95% CI: 2.10–2.78), while three regions saw a decrease, notably Central Asia, with an AAPC of −1.21 (95% CI: −1.49 to −0.92) (Table 1). In terms of individual countries and territories, AYA CRC incidence increased in all 204 regions, with the highest ASIR in Monaco (7.04; 95% CI: 0.05–57.21), followed by the United States Virgin Islands (6.88; 95% CI: 0.61–29.94) in 2021 (Figure 2).

**Table 1:** Incidence of CRC in 15–39 years between 1990 and 2021 at the global and regional level.

Location	1990		2021		AAPC‐Info	
	Number (95% UI) 1990	Rate per 100,000 population (95% UI)	Number (95% UI) 2021	Rate per 100,000 population (95% UI)	AAPCs% (95% CI) 1990–2021	*p* value
Global	41383.93 (37652.74, 44500.13)	2.02 (2, 2.04)	70200.72 (64050.21, 76855.3)	2.28 (2.26, 2.3)	0.35 (0.1, 0.61)	0.007
Sex						
Male	22362.85 (18879.24, 24506.58)	2.15 (2.13, 2.18)	42655.95 (37276.96, 48228.02)	2.74 (2.71, 2.77)	0.78 (0.46, 1.1)	0
Female	19021.08 (16813.81, 21387.59)	1.88 (1.85, 1.91)	27544.77 (24758.44, 30647.24)	1.81 (1.79, 1.83)	−0.13 (−0.22, −0.03)	0.009
SDI						
High SDI	10620.76 (10371.35, 10848.28)	2.88 (2.82, 2.93)	14068.78 (13583.42, 14621.75)	3.47 (3.41, 3.52)	0.63 (0.39, 0.87)	0
High‐middle SDI	12586.86 (11210.1, 13727.95)	2.8 (2.75, 2.85)	20440.62 (17783.46, 23798.24)	3.92 (3.86, 3.97)	1.01 (0.54, 1.48)	0
Middle SDI	13686.07 (11883.9, 15270.57)	2.02 (1.98, 2.05)	24911.41 (21708.13, 28036.36)	2.5 (2.47, 2.53)	0.69 (0.25, 1.13)	0.002
Low‐middle SDI	3365.17 (2882.08, 3893.03)	0.84 (0.81, 0.87)	7930.91 (6916.4, 9525.31)	1.03 (1.01, 1.05)	0.67 (0.47, 0.87)	0
Low SDI	1087.02 (875, 1285.3)	0.68 (0.64, 0.72)	2798.26 (2409.65, 3288.09)	0.72 (0.69, 0.75)	0.15 (0.05, 0.26)	0.003
Region						
Andean Latin America	138.01 (117.33, 160.2)	1.01 (0.85, 1.2)	405.92 (326.51, 501.6)	1.51 (1.36, 1.66)	1.26 (0.82, 1.69)	0
Australasia	266.38 (239.41, 294.37)	3.15 (2.78, 3.55)	514 (441.12, 595.18)	4.36 (3.99, 4.76)	1 (0.4, 1.6)	0.001
Caribbean	287.02 (259.67, 312.14)	2.16 (1.92, 2.43)	476.41 (400.78, 558.56)	2.58 (2.35, 2.82)	0.54 (0.22, 0.86)	0.001
Central Asia	544.74 (514.51, 575.94)	2.04 (1.87, 2.22)	551.04 (484.23, 618.2)	1.37 (1.26, 1.49)	−1.21 (−1.49, −0.92)	0
Central Europe	1136.38 (1083.19, 1191.78)	2.22 (2.09, 2.35)	990.83 (902.11, 1079.3)	2.35 (2.21, 2.51)	0.15 (−0.26, 0.57)	0.461
Central Latin America	665.59 (640.54, 692.05)	1.11 (1.02, 1.2)	2322.43 (2072.46, 2582.74)	2.3 (2.2, 2.39)	2.44 (2.1, 2.78)	0
Central Sub‐Saharan Africa	103.35 (79.15, 130.92)	0.59 (0.48, 0.71)	306.7 (226, 423.31)	0.66 (0.58, 0.73)	0.33 (0.19, 0.46)	0
East Asia	17041.66 (14143.01, 19644.4)	3.21 (3.16, 3.26)	29325.72 (24051.02, 35501.07)	5.11 (5.05, 5.17)	1.48 (0.86, 2.11)	0
Eastern Europe	2213.61 (2113.96, 2319.07)	2.32 (2.22, 2.42)	1986.12 (1824.3, 2170.77)	2.32 (2.22, 2.43)	−0.09 (−1.09, 0.92)	0.862
Eastern Sub‐Saharan Africa	514.09 (406.03, 600.9)	0.86 (0.79, 0.94)	1351.63 (1123.85, 1720.15)	0.9 (0.85, 0.95)	0.15 (0.02, 0.27)	0.019
High‐income Asia Pacific	2016.89 (1924.89, 2104.32)	2.94 (2.81, 3.07)	1904.4 (1755.35, 2051.42)	3.24 (3.1, 3.4)	0.34 (0.08, 0.61)	0.011
High‐income North America	3824.29 (3728.09, 3916.11)	3.09 (2.99, 3.19)	5450.31 (5213.79, 5649.15)	4.11 (4, 4.22)	0.96 (0.58, 1.34)	0
North Africa and Middle East	1804.18 (1419.87, 2132.9)	1.56 (1.49, 1.63)	5022.71 (4313.48, 5717.61)	1.88 (1.83, 1.94)	0.59 (0.4, 0.79)	0
Oceania	20.77 (13.81, 26.44)	0.88 (0.54, 1.37)	45.08 (34.29, 55.99)	0.84 (0.61, 1.13)	−0.12 (−0.31, 0.06)	0.196
South Asia	2405.32 (2107.57, 2737.37)	0.62 (0.59, 0.64)	5252.35 (4440.49, 6833.79)	0.68 (0.66, 0.7)	0.27 (−0.11, 0.65)	0.166
Southeast Asia	2826.78 (2279.18, 3295.53)	1.59 (1.53, 1.65)	6089.53 (5078.56, 7043)	2.13 (2.08, 2.18)	0.94 (0.8, 1.08)	0
Southern Latin America	322.62 (287.4, 359.15)	1.75 (1.56, 1.95)	717.8 (598.61, 845.99)	2.67 (2.48, 2.88)	1.43 (0.95, 1.92)	0
Southern Sub‐Saharan Africa	263.92 (240.25, 289.25)	1.44 (1.27, 1.63)	509.88 (437.37, 633.04)	1.46 (1.33, 1.59)	0.07 (−1.1, 1.26)	0.903
Sub‐Saharan Africa ‐ WB	NA	0.74 (0.69, 0.78)	NA	0.79 (0.76, 0.82)	0.2 (−0.05, 0.45)	0.117
Tropical Latin America	761.16 (725.68, 799.76)	1.29 (1.2, 1.38)	1999.25 (1874.89, 2135.54)	2.1 (2.01, 2.19)	1.5 (1.26, 1.74)	0
Western Europe	3983.05 (3822.52, 4143.86)	2.68 (2.59, 2.76)	4178.49 (3925.31, 4443.03)	2.86 (2.77, 2.95)	0.16 (−0.18, 0.51)	0.354
Western Sub‐Saharan Africa	244.11 (200.91, 290.7)	0.41 (0.36, 0.46)	800.12 (579.13, 1047.25)	0.5 (0.47, 0.54)	0.68 (0.49, 0.88)	0

Abbreviations: AAPCs, average annual percent changes; ASIR, age‐standardized incidence rate; CI, confdence interval; SDI, socio‐demographic index; UI, uncertainty interval.

### 3.2 AYA CRC Prevalence

The age‐standardized prevalence rate (ASPR) of AYACRC increased from 11.23 (95% CI: 11.18–11.27) in 1990 to 14.6 (95% CI: 14.56–14.65) in 2021, reflecting an AAPC of 0.83 (95% CI: 0.56–1.10). AYA CRC prevalence increased from 1990 to 2021 across all five SDI regions with the largest rise observed in the high‐middle SDI group, which showed an AAPC of 1.68 (95% CI: 1.37–1.99) (Figure 1 and Supporting Information S5: Table S1). The high‐middle SDI group also had the highest ASPR in 2021 (26.4, 95% CI: 26.26–26.54). The prevalence of AYA CRC increased in all 21 GBD regions. Andean Latin America had the highest ASPR at 9.76 (95% CI: 9.39–10.14), followed by Central Asia at 7.73 (95% CI: 7.46–8.01) in 2021. The largest increases in prevalence were seen in Central Latin America, with an AAPC of 2.83 (95% CI: 2.5–3.17), while two regions, including Central Asia, experienced a decrease in prevalence, with Central Asia showing an AAPC of −0.95 (95% CI: −1.24 to −0.65) (Supporting Information S5: Table S1). The AYA CRC prevalence rose in all 204 countries and territories. The highest ASPR was recorded in Monaco at 50.6 (95% CI: 16.8–121.6), followed by the United States Virgin Islands at 41.89 (95% CI: 20.25–78.19) in 2021 (Supporting Information S1: Figure S1).

### 3.3 AYA CRC Mortality

**Figure 3:**
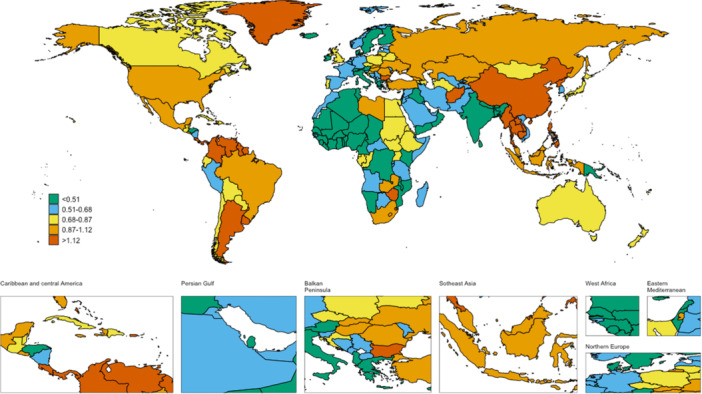
The global disease burden of AYA CRC mortality rate for both sexes in 204 countries and territories.

A slight increase in AYA CRC mortality was observed globally, with deaths rising from 23,493.06 (95% CI: 21,058.19–25,541.51) in 1990 to 26,222.38 (95% CI: 23,865.38–28,552.81) in 2021. However, the age‐standardized mortality rate (ASMR) decreased from 1.14 (95% CI: 1.13–1.16) to 0.85 (95% CI: 0.84–0.86) per 100,000 persons, reflecting an AAPC of −1.00 (95% CI: −1.29 to −0.70). The high‐middle SDI regions showed the highest ASMR at 1.19 (95% CI: 1.16–1.22) in 2021. The trend of AYA CRC mortality decreased across all five SDI regions, with the largest decline in the high‐middle SDI group at an AAPC of −0.95 (95% CI: −1.51 to −0.38) (Figure 1 and Supporting Information S6: Table S2). Among the 21 GBD regions, East Asia recorded the highest ASMR at 1.57 (95% CI: 1.54–1.60), followed by Southeast Asia at 1.17 (95% CI: 1.13–1.21) in 2021. Mortality trends decreased in 16 GBD regions, with the largest decline in Central Asia (AAPC: −1.78, 95% CI: −2.01 to −1.55). Conversely, mortality increased in five regions, with the highest increase seen in Central Latin America (AAPC: 1.19, 95% CI: 0.86–1.51) (Supporting Information S6: Table S2). At the country level, the United States Virgin Islands and Thailand exhibited the highest ASMRs, at 2.06 and 2.00, respectively (Figure 3).

### 3.4 AYA CRC DALYs

The age‐standardized DALYs rate (ASDR) decreased from 66.87 (95% CI: 66.76–66.98) to 50.19 (95% CI: 50.11–50.27) per 100,000 persons, which showed an AAPC of −0.94 (95% CI: −1.29 to −0.59) (Figure 1 and Supporting Information S7: Table S3). In the 21 GBD regions, East Asia and Southeast Asia had the highest ASDRs, at 93.19 and 68.95, respectively. Consistent with the trends in mortality, Central Asia showed the largest reduction in DALYs (AAPC = −1.79; 95% CI: −2.02 to −1.56), while Central Latin America experienced an increase (AAPC = 1.18; 95% CI: 0.88–1.49) (Supporting Information S7: Table S3). At the country level, the United States Virgin Islands had the highest ASDR at 124.35 (95% CI: 83.86–178.82), followed by Thailand at 117.63 (95% CI: 116.22–119.04) in 2021 (Supporting Information S2: Figure S2).

### 3.5 AYA CRC Burden Across Age Groups and Genders

**Figure 4:**
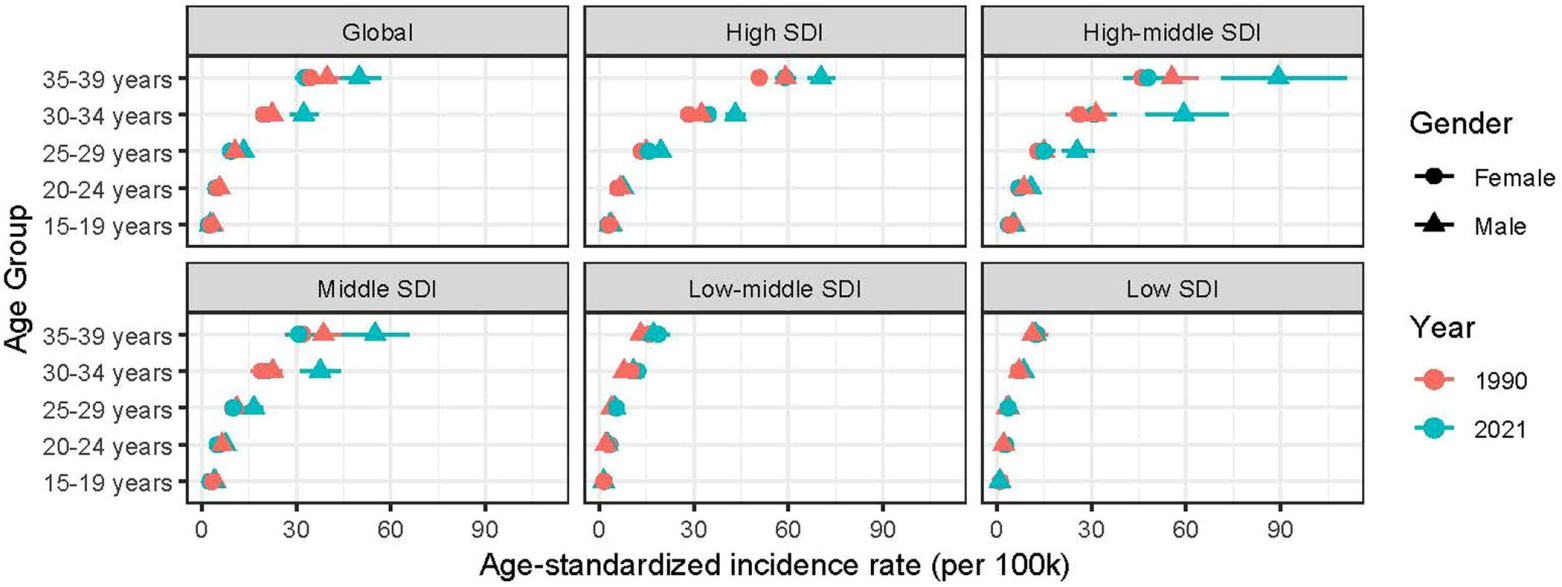
Age‐standardized incidence rate of AYA CRC globally and across 5 SDI regions in 1990 and 2021. The horizontal error bars indicate 95% confidence intervals (CIs).

**Figure 5:**
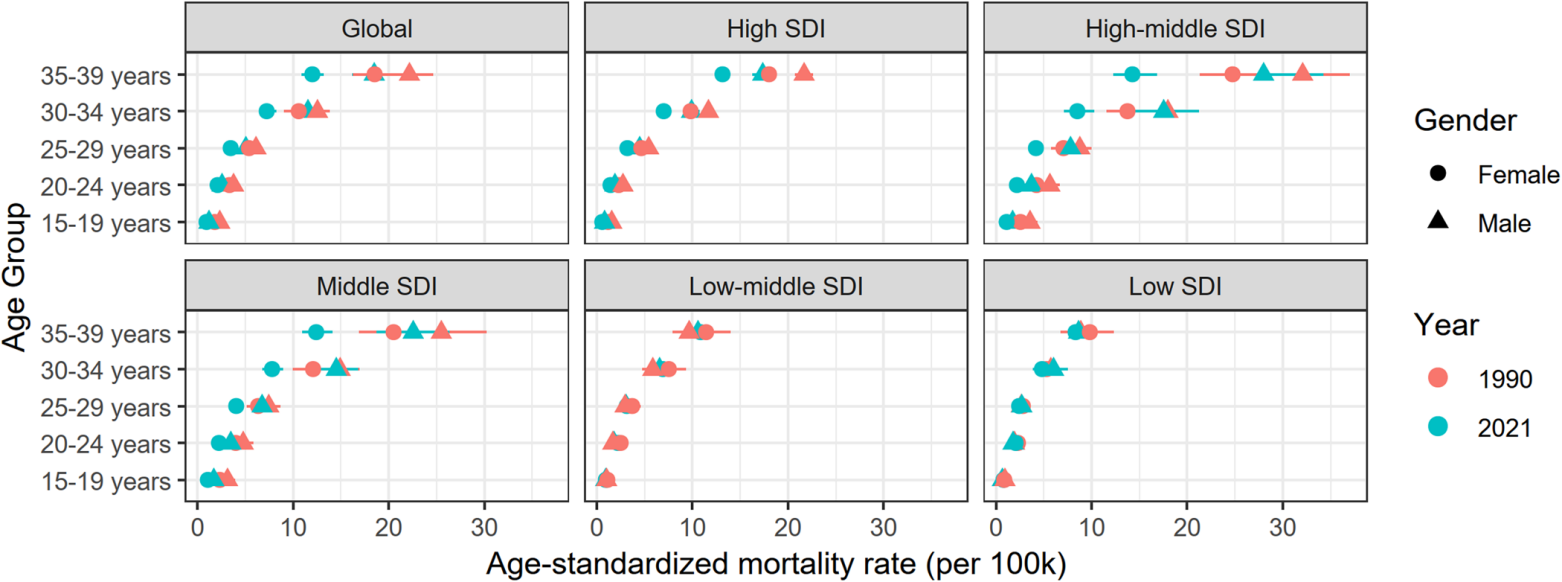
Age‐standardized mortality rate of AYA CRC globally and across 5 SDI regions in 1990 and 2021. The horizontal error bars indicate 95% confidence intervals (CIs).

Globally, the incidence and prevalence rates of AYA CRC peaked among individuals aged 35–39 years, with mortality and DALYs rates also reaching their highest levels in this age group (Figures 4 and 5, Supporting Information S3: Figure S3 and Supporting Information S4: Figure S4). Notably, males showed significantly higher rates of incidence, prevalence, DALYs, and mortality compared to females across all SDI regions in both 1990 and 2021. However, in low‐middle SDI regions, female incidence and prevalence rates surpassed those of males in 2021, indicating region‐specific gender disparities.

### 3.6 Attributable Risk Factors for DALYs in AYA CRC

**Figure 6:**
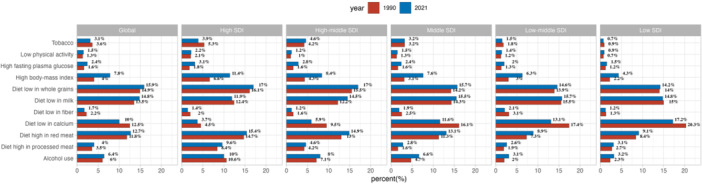
The AYA CRC DALYs attributable to risk factors compared in 1990 and 2021, globally and by region.

The analyses revealed that dietary factors were the primary contributors to AYA CRC DALYs. Specifically, low whole‐grain intake (15.9%), low milk consumption (14.8%), and high red meat consumption (12.7%) emerged as the top three risk factors globally in 2021. This pattern varied by SDI: high‐middle SDIs were primarily influenced by low whole‐grain intake, while low SDIs saw higher contributions from low calcium intake. Additionally, a high BMI emerged as a significant risk factor, showing a notable increase from 1990 to 2021 across global and all SDI regions (Figure 6).

### 3.7 Prediction of Future Burden of AYA CRC

**Figure 7:**
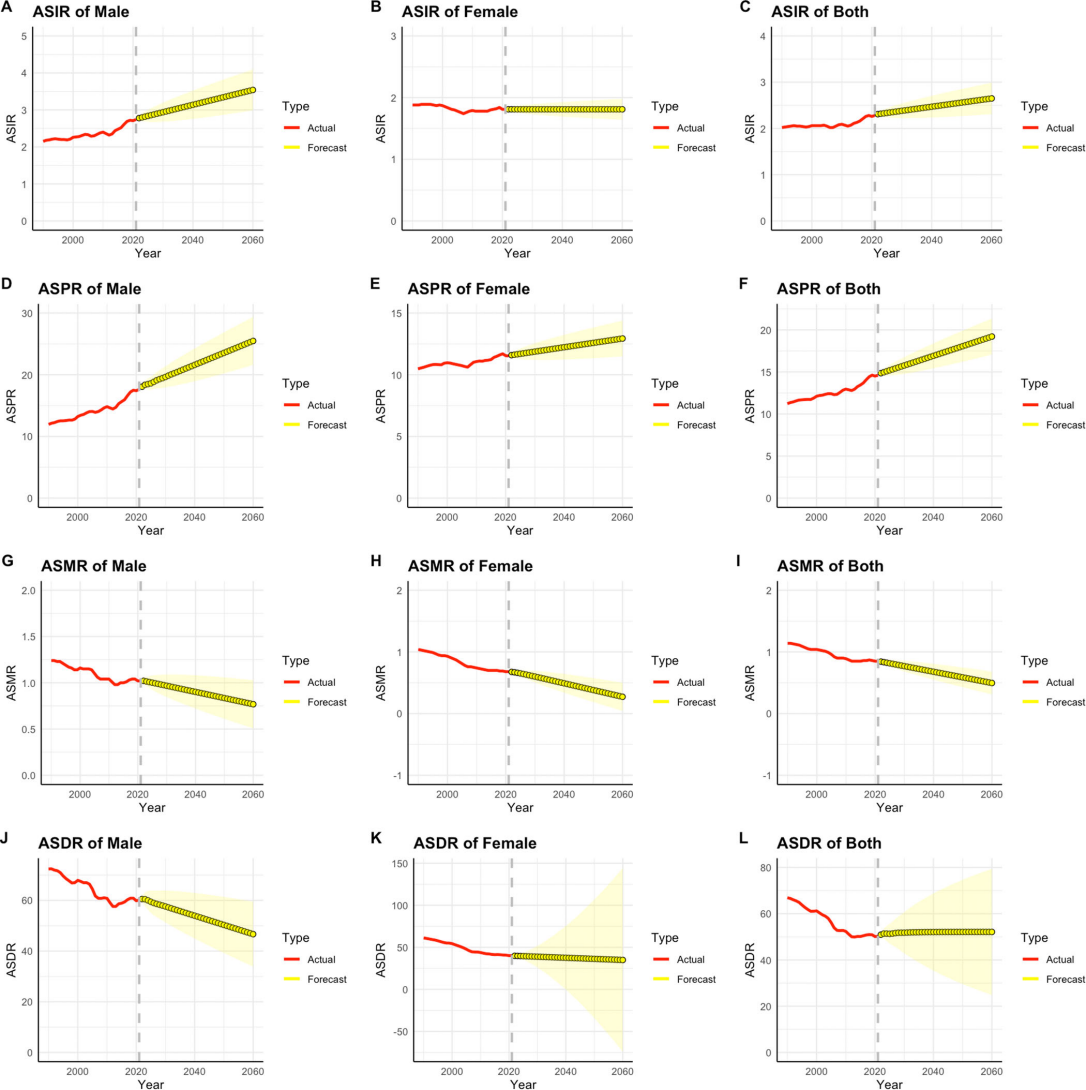
Future forecasts of GBD in AYA CRC incidence, prevalence, DALYs and Mortality to 2060. Red lines represent actual data, and yellow lines with shaded regions denote forecasted trends with 95% confidence intervals (CIs).

Figure 7 illustrates the projected trends in global AYA CRC incidence, indicating a steady rise in the number of affected individuals. The ASIR for global AYA CRC is projected to reach 2.65 (95% CI: 2.31–2.998) per 100,000 population by 2060. The expected ASIR for men (3.54, 95% CI: 2.99–4.09) by 2060, is significantly higher than for women (1.81, 95% CI: 1.64–1.98). Similar trends are expected for global AYA CRC prevalence, projected to reach 19.21 per 100,000 people (95% CI: 17.07, 21.35) by 2060, with men at 25.49 (95% CI: 21.59, 29.38) and women at 12.94 (95% CI: 11.49, 14.39). The ASMR for global AYA CRC is projected to reach 0.50 per 100,000 population (95% CI: 0.31, 0.68) by 2060, with men at 0.77 (95% CI: 0.51, 1.03) and women at 0.27 (95% CI: 0.04, 0.50), showing a significant decrease. The ASDR for global AYA CRC is projected to reach 52.12 (95% CI: 24.85, 79.39) per 100,000 population by 2060, with men at 46.67 (95% CI: 33.87, 59.48) and women at 34.96 (95% CI: −74.36, 144.28).

## 4 Discussion

Based on data from the GBD 2021, this study presents the latest insights into the epidemiological burden of colon and rectum cancer among adolescents and young adults. The findings highlight a significant global increase in both the incidence and prevalence of AYA CRC, while mortality and DALYs have notably decreased from 1990 to 2021. These trends are consistent with previous studies, which have reported rising incidence and prevalence, alongside declining mortality and DALYs across all age groups worldwide [21].

The highest AISR, ASPR, ASDR, and ASMR in 2021 were observed in high‐middle SDI regions. Similar patterns were seen among males. However, in females, the highest AISR and ASPR in 2021 were found in high‐SDI regions. These results underscore the ongoing socioeconomic disparities in the global burden of AYA CRC. Several factors, including obesity, diet, sedentary behavior, and consumption of processed foods, red meat, and sugary drinks, contribute to this higher burden [22]. In East Asia, increasing urbanization, westernized diets, and globalization may partly explain the growing burden of AYA CRC due to their associated environmental, lifestyle, and metabolic risk factors [23]. Genetic factors may also contribute to elevated CRC risk. However, adopting a healthier lifestyle can potentially mitigate the genetic predisposition to CRC. Notably, the greatest increase in AYA CRC mortality and DALYs occurred in Central Latin America, where socioeconomic inequalities are considered a major contributor to premature mortality [24]. In contrast, the most significant decline in AYA CRC mortality and DALYs from 1990 to 2021 was seen in Central Asia. In countries with active screening programs, such as Kazakhstan and Lithuania, the decline in CRC mortality has been more pronounced [25].

The incidence of AYA CRC has risen in all 204 countries and territories, with the highest ASIR recorded in Monaco, the United States Virgin Islands, and Puerto Rico in 2021. These findings align with previous studies that identified Monaco, the Netherlands, and Bermuda as the top three countries with the highest CRC incidence across all age groups [21]. In terms of mortality, the highest ASMR and ASDR in 2021 were observed in the United States Virgin Islands, Thailand, and the Bahamas. However, Uruguay, Hungary, and Bulgaria had the highest CRC mortality rates across all age groups [21].

This study also found that the incidence of AYA CRC has generally increased, peaking in the 35–39 age group, especially in the high‐SDI regions and high‐middle‐SDI regions. This is consistent with reports from Wang et al., who noted that CRC incidence increases in Europe over the past 25 years before the age of 40, with the most significant rise observed in individuals aged 20–39 years [26]. These findings underscore the need for advanced screening strategies in high‐SDI and high‐middle‐SDI regions to mitigate the rising burden. Specifically, for average‐risk individuals, we align with current guidelines recommending initiation of CRC screening at age 45 years and continuation through age 75 years [27]. High‐risk populations—defined here as those with a first‐degree family history of CRC (e.g., parent, sibling, or child diagnosed at any age) or obesity (BMI ≥ 30 kg/m^2^)—warrant earlier intervention. Integrating our observation of burden peaking in the 35–39 age group, we propose considering screening initiation as early as age 35 years for these high‐risk groups, or 10 years before the youngest family member's diagnosis if earlier, to capture the pre‐peak escalation in incidence [28]. However, the cost‐effectiveness of this aggressive screening recommendations needs further study validation.

As in previous studies, we also identified a diet low in whole grains, a low intake of milk, and a high consumption of red meat as major risk factors for AYA CRC mortality globally. Increasing the intake of whole grains and milk while reducing red meat consumption could help reduce the global burden of AYA CRC. Additionally, the association between elevated BMI and CRC risk has become more pronounced over time. This trend aligns with global epidemiological data showing that higher BMI is strongly linked to an increased risk of CRC, particularly in regions with higher socioeconomic status [29, 30]. The more significant association between high BMI and AYA CRC in high‐SDI regions may be driven by urbanization‐related lifestyle changes. The obesogenic environments—characterized by diets high in processed red meats, refined sugars, and saturated fats, coupled with sedentary lifestyles and reduced physical activity—exacerbate adiposity‐related carcinogenesis [31]. These promote chronic low‐grade inflammation, insulin resistance, and hyperinsulinemia, which enhance colonic epithelial proliferation and adenoma formation via pathways like IGF‐1 signaling and adipokine dysregulation (e.g., elevated leptin and reduced adiponectin) [31].

We projected the disease burden of AYA CRC from 2022 to 2060, anticipating a 14.7% increase in the ASIR. The ASIR among males is expected to rise by 27.4%, significantly higher than the increase observed in females. These projections align with previous research, which suggests the urgent need for CRC screening programs, such as fecal occult blood testing (FOBT) and colonoscopy, especially among males, to prevent the occurrence of CRC [32]. Furthermore, by 2060, the ASMR is projected to decrease by 41.1%, which may be partly attributed to effective public health interventions and advancements in cancer treatments. However, the ARIMA model's predictions rely on the assumption of ‘no major intervention breakthroughs, limiting its ability to account for external shocks or policy shifts that could alter trends [33]. If future public health interventions (e.g., widespread promotion of HPV vaccines—as studies have established a significant oncogenic role for HPV in CRC pathogenesis, with infection conferring up to sevenfold increased risk—or novel screening technologies (e.g., blood ctDNA‐based early detection), are scaled up, the actual AYA CRC burden may be lower than our projections [34, 35]. Additionally, unforeseen public health emergencies (e.g., pandemics)—as evidenced by COVID‐19's significant drop in CRC screening rates, leading to delayed diagnoses and advanced‐stage shifts—could disrupt historical trends and affect prediction accuracy [36, 37]. Furthermore, during model construction, we systematically tested different ARIMA parameters (*p* = 1–3, *d* = 0–1, *q* = 1–3) and the sensitivity analysis showed that adjustments to parameters resulted in a variation coefficient of < 10% for ASIR predictions—indicating minimal impact on results.

Several important limitations should be considered in this study. First, the age threshold for CRC in the GBD 2021 was set at 15 years, so children and adolescents under 15 were not included in our analysis. The incidence of CRC in this younger age group is rare, and we believe that including them would not have significantly altered the main findings. Second, the quality and availability of data vary considerably across regions and countries. Less developed nations often lack comprehensive, population‐based cancer registries, which can result in misdiagnosis or underdiagnosis of the true AYA CRC burden. Third, while this study includes several known risk factors for AYA CRC, the list is not exhaustive. Since all data is based on the GBD 2021 study, other potential risk factors such as genetic susceptibility and environmental pollutants could not be assessed. Last but the not the least, for the risk factor analysis, although subgroup analyses for core risk factors might enhance the precision of intervention targets, we did conduct is considering that the GBD 2021 dataset has limited data integrity for PAF estimates in narrow age subgroups of AYAs. For younger age groups (e.g., 15–19 years), the number of CRC cases is extremely small, leading to unstable PAF estimates with wide 95% UIs. Such results may be misleading and fail to provide reliable guidance for intervention targets.

In conclusion, this study demonstrates a substantial global increase in the incidence and prevalence of AYA CRC from 1990 to 2021, accompanied by a notable decline in mortality and DALYs. These findings advocate for region‐specific interventions, such as subsidized screening programs in low‐SDI regions and public health campaigns targeting processed food consumption in urbanized areas to reduce the global burden of AYA CRC. Screening colonoscopy should be appropriately advanced for high‐risk individuals.

## Author Contributions

Ling Zhao and Ning‐Bo Hao designed the study. Ya‐Fei He, Lu Zhang, and Dun‐Zhong Hu collected data. Ling Zhao, Ning‐Bo Hao, Chang‐Zheng Li, Dun‐Zhong Hu, and Yan‐Ni Li analyzed the data. Ya‐Fei He, Lu Zhang, and Dun‐Zhong Hu wrote the manuscript. All authors have read and approved the final version of the manuscript.

## Disclosure

The corresponding author Ling Zhao had full access to all of the data in this study and takes complete responsibility for the integrity of the data and the accuracy of the data analysis.

## Conflicts of Interest

The authors declare no conflicts of interest.

## Transparency Statement

The lead author Ning‐Bo Hao, Ling Zhao affirms that this manuscript is an honest, accurate, and transparent account of the study being reported; that no important aspects of the study have been omitted; and that any discrepancies from the study as planned (and, if relevant, registered) have been explained.

## Supporting information


**Figure S1:** The global disease burden of AYA CRC Prevalence rate for both sexes in 204 countries and territories.

**Figure S2:** The global disease burden of AYA CRC DALYs rate for both sexes in 204 countries and territories.

**Figure S3:** Age‐specific prevalence rate of AYA CRC globally and across 5 SDI regions in 1990 and 2021.

**Figure S4:** Age‐standardized DALY rate of AYA CRC globally and across 5 SDI regions in 1990 and 2021.

**Table S1:** prevalence of CRC in 15 to 39 years between 1990 and 2021 at the Global and Regional Level.

**Table S2:** Mortality of CRC in 15 to 39 years between 1990 and 2021 at the Global and Regional Level.

**Table S3:** DALYs of CRC in 15 to 39 years between 1990 and 2021 at the Global and Regional Level.

## Data Availability

The authors confirm that all the data supporting the article are available in a public repository with the link: https://www.alipan.com/s/QQgKX9ZDALX.
