# Supplementary figures and images for: Global Burden of Colorectal Cancer in Adolescents and Young Adults From 1990 to 2021: A Systematic Analysis of the Global Burden of Disease Study 2021
Source: Health Sci Rep. 2025 Nov 30;8(12):e71587. doi: 10.1002/hsr2.71587 (PMC12665505; doi:10.1002/hsr2.71587)

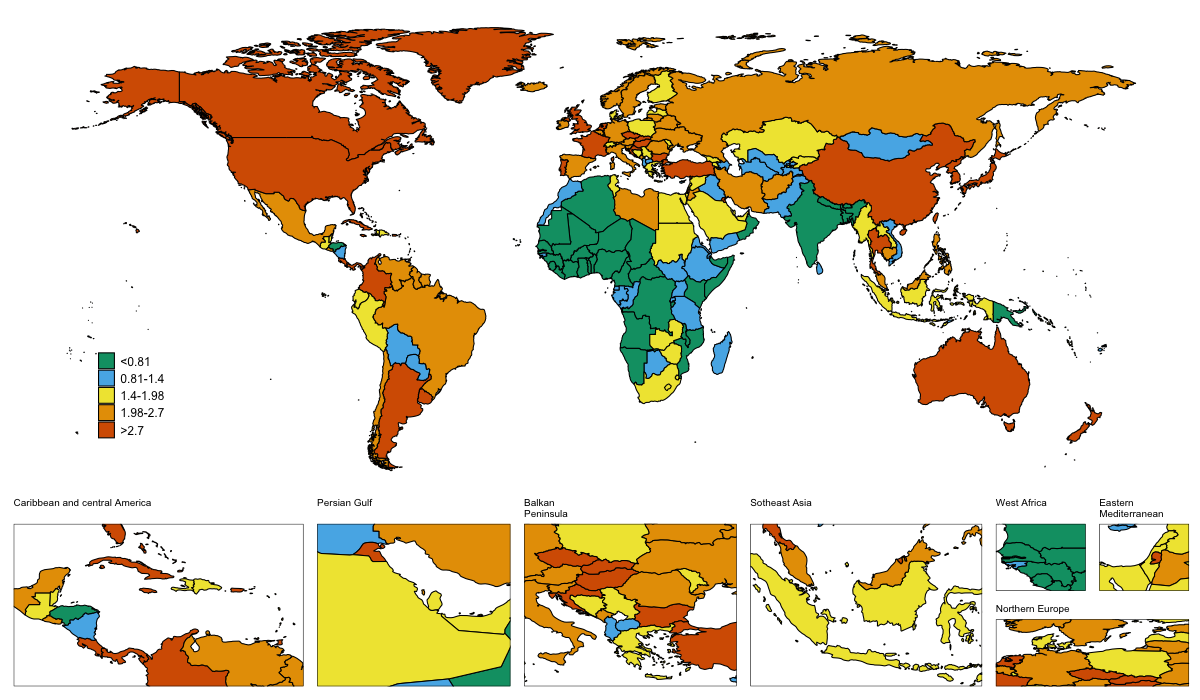

Supplement: Supplementary file 1 — Figure S1: The global disease burden of AYA CRC Prevalence rate for both sexes in 204 countries and territories. [file HSR2-8-e71587-s005.tiff]

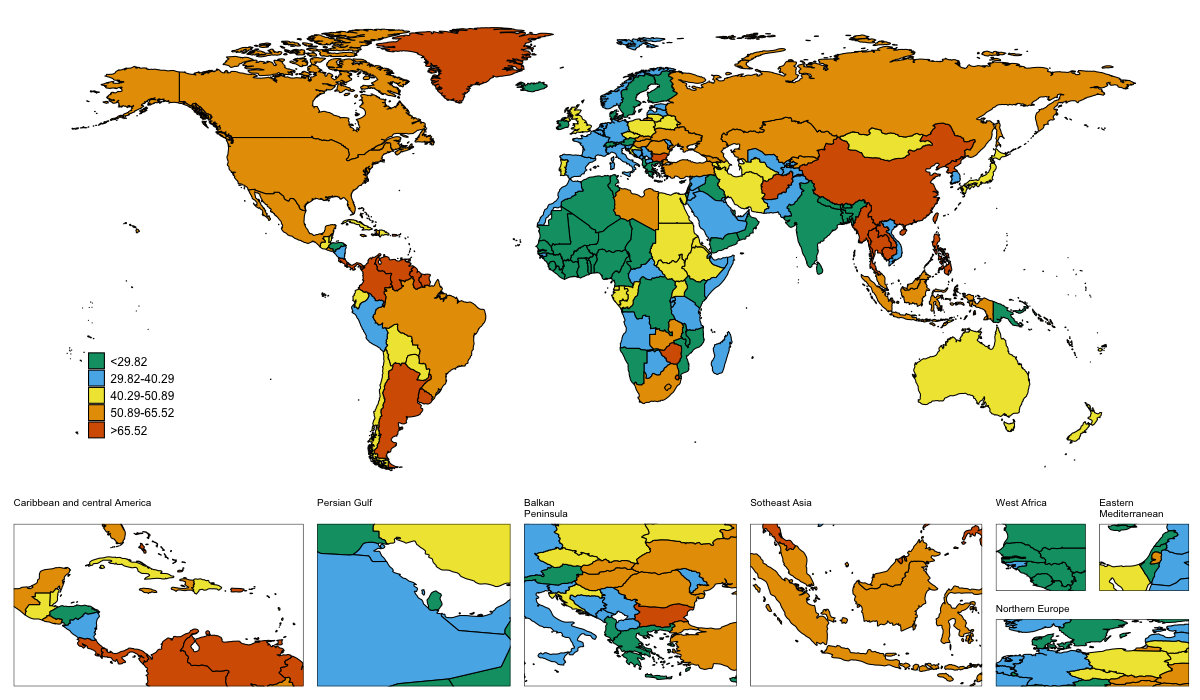

Supplement: Supplementary file 2 — Figure S2: The global disease burden of AYA CRC DALYs rate for both sexes in 204 countries and territories. [file HSR2-8-e71587-s003.tiff]

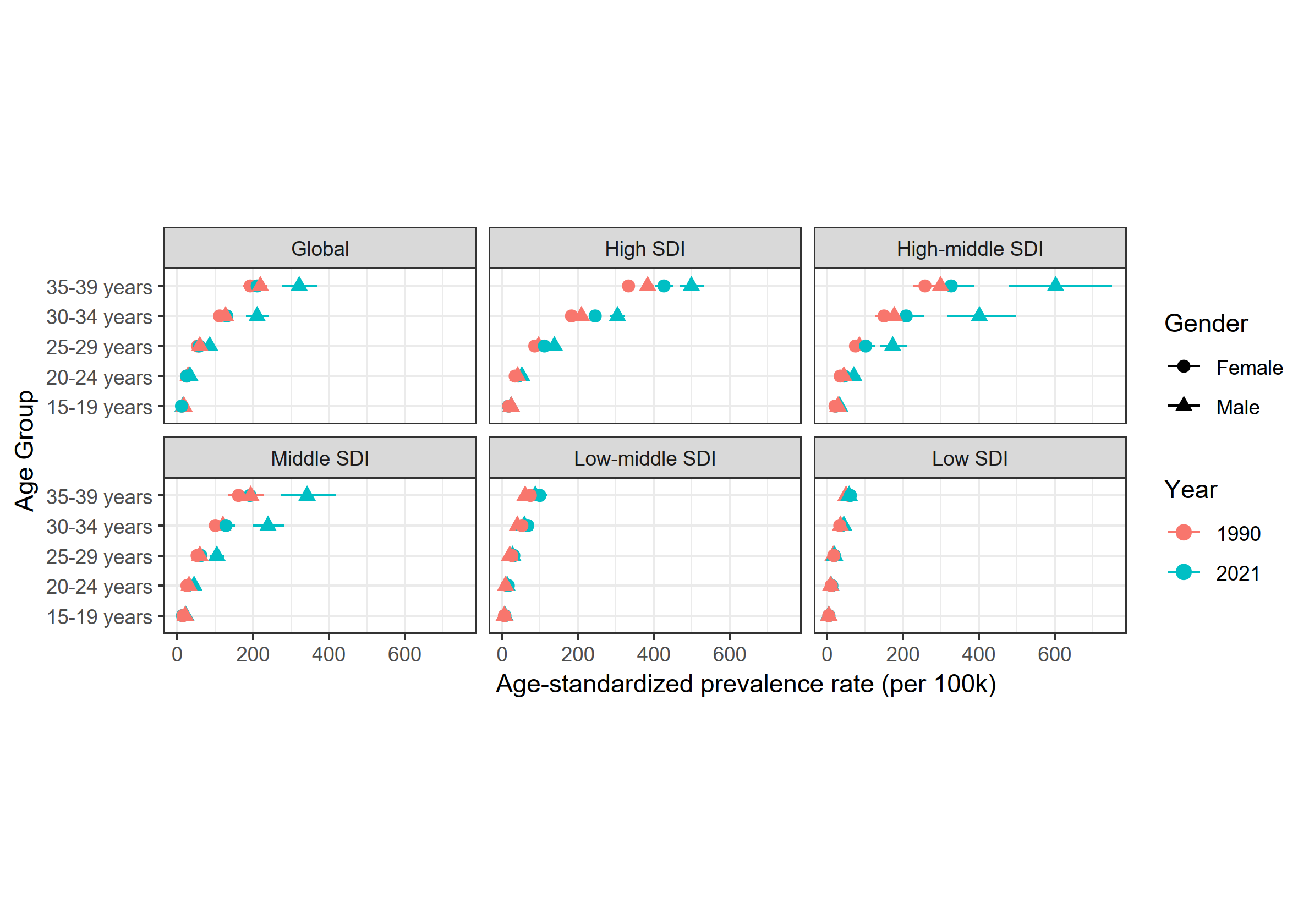

Supplement: Supplementary file 3 — Figure S3: Age‐specific prevalence rate of AYA CRC globally and across 5 SDI regions in 1990 and 2021. [file HSR2-8-e71587-s007.tif]

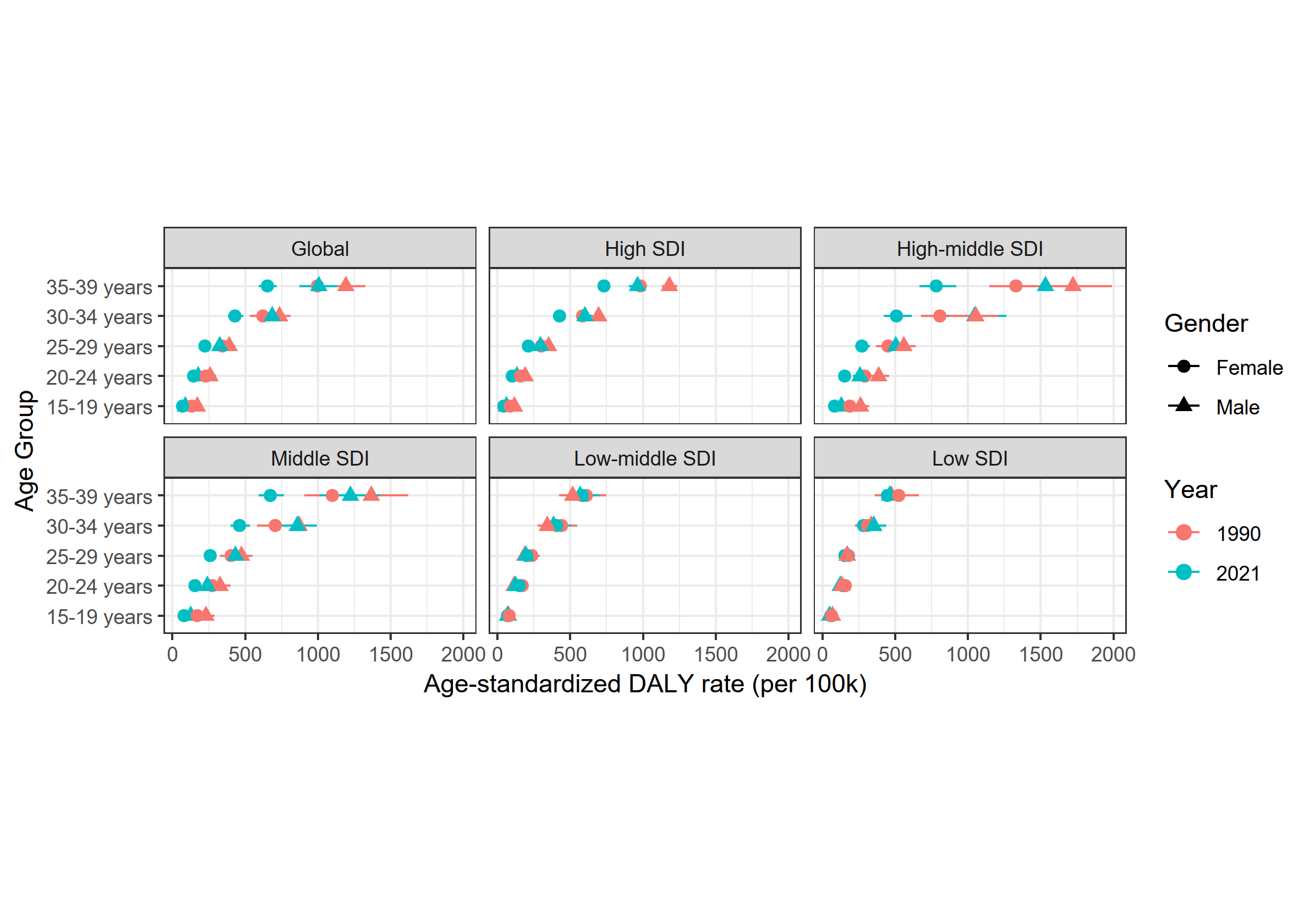

Supplement: Supplementary file 4 — Figure S4: Age‐standardized DALY rate of AYA CRC globally and across 5 SDI regions in 1990 and 2021. [file HSR2-8-e71587-s006.tif]
